# Hospitalization for hypoglycaemia in people with diabetes in Denmark, 1997–2017: Time trends in incidence and HbA_1c_ and glucose‐lowering drug use before and after hypoglycaemia

**DOI:** 10.1002/edm2.227

**Published:** 2021-01-21

**Authors:** Mads Bisgaard Bengtsen, Jakob Schöllhammer Knudsen, Maria Bisgaard Bengtsen, Niels Møller, Reimar Wernich Thomsen

**Affiliations:** ^1^ Department of Endocrinology and Internal Medicine Aarhus University Hospital Aarhus Denmark; ^2^ Department of Internal Medicine Regional Hospital of Horsens Horsens Denmark; ^3^ Department of Clinical Epidemiology Institute of Clinical Medicine Aarhus University Hospital Aarhus Denmark; ^4^ Department of Clinical Pharmacology Aarhus University Hospital Aarhus Denmark

**Keywords:** glucose‐lowering drugs, hospitalization for hypoglycaemia, hypoglycaemia, type 1 diabetes, type 2 diabetes

## Abstract

**Objective:**

To assess incidence trends of first hospitalization for hypoglycaemia in Denmark and to examine HbA_1c_ levels and glucose‐lowering drug use before and after hospitalization among individuals with type 1 or type 2 diabetes.

**Research Design and Methods:**

We performed a population‐based study linking diagnosis, prescription and laboratory data. Standardized incidence of first hospitalization for hypoglycaemia in Denmark was assessed for each calendar year 1997–2017. HbA1c and glucose‐lowering drug use was compared with age‐ and sex‐matched diabetes comparisons without hospitalization for hypoglycaemia.

**Results:**

The annual age‐ and sex‐standardized incidence rate of first hospitalization for hypoglycaemia per 100,000 person‐years increased during 1997–2003 (from 17.7 to 30.3 per 100,000 person‐years), remained stable until 2010 (30.4) and gradually declined until 2017 (22.0). During this period, we identified 3,479 people with type 1 diabetes and 15,329 people with type 2 diabetes experiencing first hospitalization for hypoglycaemia. Both diabetes groups experienced a mean HbA1c decrease of ~12%–15% in the months preceding first hospitalization, followed by a gradually increasing HbA1c afterwards. People with type 1 diabetes and hospitalization used similar insulin therapies as those without hospitalization. People with type 2 diabetes and hospitalization more often received insulin (55%) than comparisons (45%), and 45% discontinued insulin or stopped all glucose‐lowering therapy after first hospitalization.

**Conclusions:**

Incidence of hospitalizations for hypoglycaemia has declined by one fourth the last decade in the Danish population. A HbA1c decrease precedes first hospitalization for hypoglycaemia in individuals with diabetes, and profound changes in glucose‐lowering drug therapy for type 2 diabetes occur after hospitalization.

## Graphical abstract

The incidence of hospitalization for hypoglycaemia has declined considerably during the last decade in the Danish population. An observable HbA1c decrease precedes hospitalization, and drastic changes in glucose‐lowering drug therapy occur among people with type 2 diabetes after hospitalization

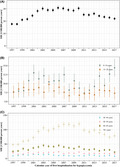

**Bulleted novelty statement**

**What is already known?**

Hospitalization for hypoglycaemia is associated with increased morbidity and mortality in people with diabetes, and severe hypoglycaemia is the strongest predictor of future events of severe hypoglycaemia.

**What this study has found?**

The incidence of hospitalization for hypoglycaemia has declined during the last decade in the Danish population. An observable HbA_1c_ decrease precedes hospitalization, and treatment discontinuation is frequent among people with type 2 diabetes after first hospitalization.

**What are the clinical implications of the study?**

The declining incidence of hospitalization for hypoglycaemia is likely caused by a number of improvements in diabetes management. The observed decline in HbA1c preceding hospitalization for hypoglycaemia may reflect intensification of treatment and implies that good monitoring is important when intensifying pharmaceutical therapy.

## 1 INTRODUCTION

Hypoglycaemia is a common and feared condition in people with type 1 and type 2 diabetes, remaining a hindrance for optimal glycemic management. Consequently, some people intentionally reduce doses or discontinue glucose‐lowering drugs trying to prevent episodes of hypoglycaemia,^1^ possibly leading to HbA_1c_ dysregulation.^1^ While non‐severe episodes of hypoglycaemia are self‐manageable, severe hypoglycaemia requires external assistance and possibly leads to hospitalization. Hospitalization for hypoglycaemia represents a tremendous economic burden, and treatment in hospital setting is several fold higher than in community setting.^1^ Hospitalization for hypoglycaemia is associated with increased morbidity and mortality, ^1^ and the first event is the strongest predictor of future events of severe hypoglycaemia.^2^

Recent years have witnessed newer glucose‐lowering drugs being introduced to the market, and increased availability of technological devices designed to improve diabetes treatment (eg insulin pump, continuous glucose monitoring).^3^ However, the extent to which these changes in diabetes management correlate with reduced incidence of hospitalization for hypoglycaemia in adults with type 1 and type 2 diabetes is unclear. Previous studies examining incidence rates of hospitalization for hypoglycaemia have reported opposing results,^4^, ^5^, ^6^, ^7^, ^8^ and few studies have focused on the first event. Understanding the current incidence trends of first hospitalization for hypoglycaemia in real‐world diabetes populations and understanding which individuals are at increased risk is essential.

We aimed to assess: 1) incidence trends in first hospitalization for hypoglycaemia 1997–2017 in the country of Denmark and 2) trends in HbA_1c_ and glucose‐lowering drug use before and after first hospitalization for hypoglycaemia in people with type 1 diabetes and type 2 diabetes.

## 2 RESEARCH DESIGN AND METHODS

### 2.1 Setting

We conducted a population‐based study based using prospectively collected health care data. The Danish National Health Service provides universal, tax‐funded healthcare, guaranteeing access to primary and secondary sectors and partial reimbursement for prescribed drugs. The unique personal registry number assigned to all Danish residents at birth or immigration allows for unambiguous data linkage.^9^ We linked several existing population‐based medical databases, as described below. The Danish National Prescription Registry was established 1994 covering all redeemed prescriptions at pharmacies in Denmark.^10^ The Danish National Patient Registry (DNPR) contains information on admissions and discharges from all Danish non‐psychiatric hospitals since 1977. Since 1995, records of emergency and outpatient specialist clinic visits are included.^11^ The coding used is the *International Classification of Diseases*, *Eighth Revision* (ICD‐8) until the end of 1993 and *Tenth Revision* (ICD‐10) thereafter. Complete laboratory results from tests ordered in primary, secondary and tertiary care facilities in Northern Denmark (1.8 million people) have been recorded since 2000 in the Clinical Laboratory Information System (LABKA) database. Incomplete data are available in LABKA for the 1985–1999 period.^12^

### 2.2 Study population: Incidence

From the DNPR, we identified all people with a first hospitalization for hypoglycaemia (using ICD‐10 codes: E100, E110, E120, E130, E140, E159, E160, E161 and E162) between 1JAN1977 and 31DEC2018. To restrict our population to people with incident hypoglycaemia hospitalization, we excluded people diagnosed with hypoglycaemia before 1997.

### 2.3 Study population: Individuals with diabetes

Similar to other studies,^13^ we identified individuals with incident diabetes using either the date of their first ever redemption of a glucose‐lowering drug prescription (Anatomical Therapeutic Chemical classification system [ATC] code starting with A10) or their first ever DNPR hospital‐coded diabetes (ICD‐8 or ICD‐10 code starting with 249–250, 2515, E10‐E15, O24, T383A, M142, G590, G632, H280, H334, H450, H360, N083), whichever came first. This algorithm for identifying diabetes has been found to have a positive predictive value (PPV) of 97% for hospital‐based diagnoses and 95% for prescription‐based diagnoses, whereas estimated sensitivity of this combined approach for detecting known diabetes is higher than 80%.^14^ We excluded individuals who had not resided in Denmark for at least one year prior to this date. To ensure a high specificity for type 1 diabetes, people who redeemed insulin before age 30 years (ATC code starting with A10A) or any glucose‐lowering drug before age 15 years were considered as likely having type 1 diabetes. Women giving birth within nine months after diabetes diagnosis were excluded as likely having gestational diabetes mellitus. Women with pre‐existing hospital diagnosed polycystic ovarian syndrome or who redeemed any metformin prescription (ATC code A10BA02) in combination with clomifene (ATC code G03 GB02) within 12 months following diagnosis were excluded as likely having polycystic ovarian syndrome. We defined the remaining people with diabetes as having type 2 diabetes.

At the time of hospitalization, we matched each individual with diabetes with three individuals with diabetes who had not experienced a hospitalization (comparisons) up to the date of the corresponding individual. Matching was performed on age (birth year in five calendar year intervals), sex and diabetes type (1 or 2) using the ccwc function from the Epi package.^15^

### 2.4 Patient characteristics

Data on sex and age at date of first hospitalization for hypoglycaemia/matched index date were obtained from the Danish Civil Registration System (CRS).^16^ Age was categorized in the following groups: [0–14 years], [15–29 years], [30–49 years], [50–59 years], [60–69 years] and [70+ years]. For people living in Northern Denmark, the latest HbA_1c_ measurement before the hospitalization for hypoglycaemia date was obtained from the LABKA database. We categorized the following pre‐treatment HbA_1c_ levels: [<48 mmol/mol], [48–52 mmol/mol], [53–57 mmol/mol], [58–63 mmol/mol], [64–74 mmol/mol], [75–85 mmol/mol] and [≥86 mmol/mol] ([<6.5%], [6.5–6.9%], [7.0–7.4%], [7.5–7.9%], [8.0–8.9%], [9.0–9.9%] and [≥10%]). We defined diabetes duration from the time of diagnosis until the time of hospitalization for hypoglycaemia. We also assessed the non‐diabetes comorbidity burden using the Charlson comorbidity index (CCI) ^17^ and calculated a total score for each patient (no comorbidities [score =0], moderate comorbidity burden [score =1], severe comorbidity burden [score =2] or very severe comorbidity burden [score >2]).

### 2.5 Treatment categorization

Treatment categories for people with type 1 diabetes were defined as: insulin pump (redeeming only prescriptions for fast‐acting insulin [ATC: A10AB]), intermediate +combined insulin (intermediate‐acting or intermediate‐ or long‐acting combined with fast‐acting (ATC: A10AC, A10AD or A10AF), long‐acting insulin (ATC: A10AE) or uncertain (none of the above). Treatment categories for people with type 2 diabetes were defined as: metformin monotherapy, metformin +other non‐insulin glucose‐lowering drug (NI‐GLD), sodium‐glucose transport protein 2 inhibitors (SGLT2) monotherapy, no treatment, glucagon‐like peptide‐1 receptor analogues (GLP‐1RA) monotherapy, dipeptidyl peptidase‐4 inhibitors (DPP4i) monotherapy, sulfonylurea (SU) monotherapy, other NI‐GLD monotherapy, other NI‐GLD combinations, insulin monotherapy, metformin +insulin, insulin +other NI‐GLD and metformin +insulin + other NI‐GLD (as described in more detail elsewhere ^18^).

### 2.6 Statistical Analysis

**FIGURE 1:**
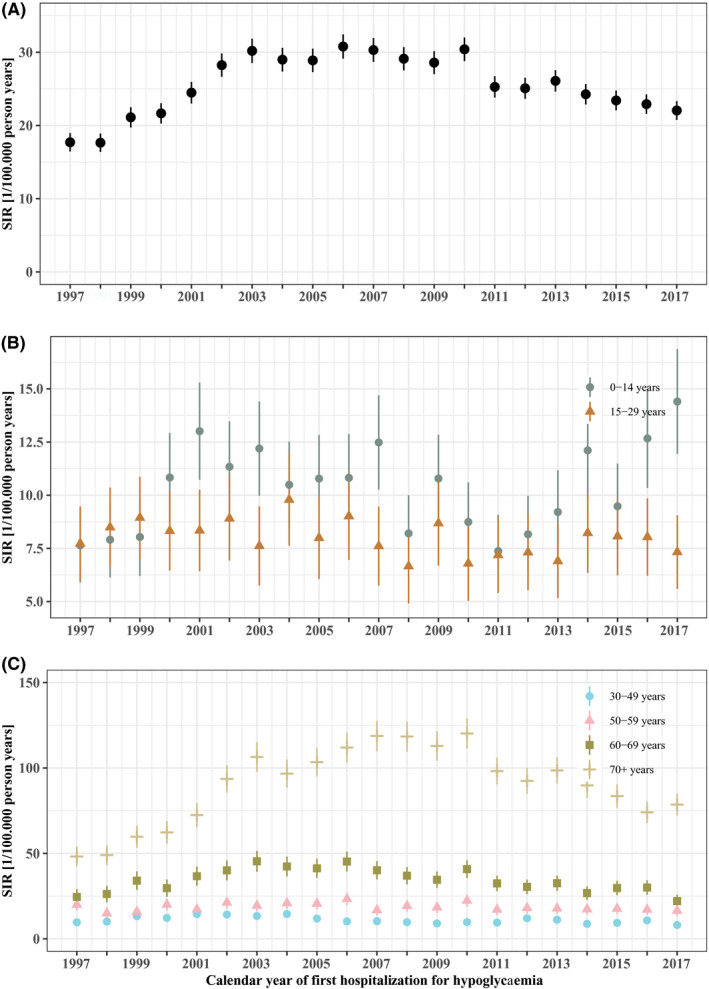
Incidence trends. A: Overall age‐ and sex‐standardized incidence rates (SIR) from 1997 to 2017 of first hospitalization for hypoglycaemia in Denmark, and B and C: SIR by age groups. Line with each SIR indicates 95% CI.

First, we computed and plotted the annual age‐ and sex‐standardized incidence rates (SIRs) of first hospitalization for hypoglycaemia in the country of Denmark during 1997–2017 (with 95% confidence intervals) overall (Supplementary Table S1) (Figure 1: A) and by age groups (Figure 1: B and C). Rates were standardized to the age distribution of the Danish population in the year 2017.

Second, we characterized people with either type 1 or type 2 diabetes hospitalized for hypoglycaemia and their comparisons at the time of hospitalization / index date (Table 1).

**TABLE 1:** Characteristics of people with diabetes and first hospitalization for hypoglycaemia in Denmark and comparisons matched on age, sex and diabetes type 1997–2017.

	Type 1 diabetes with hospitalization (N = 3,479)	Type 1 diabetes without hospitalization (N = 10,419)	Type 2 diabetes with hospitalization (N = 15,329)	Type 2 diabetes without hospitalization (N = 46,009)
Male	2,031 (58)	6,127 (59)	8,292 (54)	24,875 (54)
Female	1,448 (42)	4,292 (41)	7,037 (46)	21,134 (46)
Median Age (IQR)	30.80 (18.40, 41.85)	30.80 (18.30, 41.70)	73.70 (63.60, 81.50)	73.70 (63.70, 81.50)
0–14 years	613 (18)	1,856 (18)	0 (0)	0 (0)
15–29 years	987 (28)	2,912 (28)	0 (0)	0 (0)
30–49 years	1,426 (41)	4,316 (41)	841 (5)	2,476 (5)
50–59 years	252 (7)	740 (7)	1,671 (11)	5,012 (11)
60–69 years	51 (1)	135 (1)	2,886 (19)	8,596 (19)
70+ years	0 (0)	0 (0)	9,219 (60)	27,777 (60)
Duration median years (IQR)	7.59 (3.98, 12.62)	7.41 (3.84, 12.33)	8.53 (4.62, 13.08)	8.11 (4.38, 12.71)
no comorbidities	2,864 (82)	8,867 (85)	5,206 (34)	21,205 (46)
moderate comorbidities	239 (7)	748 (7)	3,426 (22)	10,493 (23)
severe comorbidities	184 (5)	447 (4)	2,713 (18)	7,113 (15)
very severe comorbidities	192 (6)	357 (3)	3,984 (26)	7,198 (16)

HbA_1c_ (mmol/mol; %)^a^	Type 1 diabetes with hospitalization (N = 1,108)	Type 1 diabetes without hospitalization (N = 3,077)	Type 2 diabetes with hospitalization (N = 5,848)	Type 2 diabetes without hospitalization (N = 17,010)
no measurement	269 (20)	870 (23)	861 (18)	2,566 (18)
< 48 mmol/mol; 6.5%	79 (6)	165 (4)	1,096 (23)	2,021 (14)
48–52 mmol/mol; 6.5–6.9%	88 (7)	157 (4)	584 (12)	1,683 (12)
53–57 mmol/mol; 7–7.4%	124 (9)	349 (9)	510 (11)	1,618 (11)
58–63 mmol/mol / 7.5–7.9%	151 (11)	398 (11)	421 (9)	1,519 (11)
64–74 mmol/mol; 8–8.9%	294 (22)	768 (21)	604 (13)	2,332 (17)
75–85 mmol/mol; 9–9.9%	147 (11)	478 (13)	387 (8)	1,276 (9)
86 mmol/mol; >=10%	163 (12)	538 (14)	334 (7)	1,100 (8)
Median HbA_1c_ (IQR) ^a^	1,046; 8.20 (7.37, 9.20)	2,853; 8.40 (7.50, 9.50)	3,936; 7.20 (6.40, 8.50)	11,549; 7.60 (6.72, 8.70)
no EGFR measurement ^a^	317 (24)	1,048 (28)	816 (17)	2,497 (18)
>=60 ^a^	859 (65)	2,324 (62)	1,671 (35)	5,165 (37)
<60 ^a^	139 (11)	351 (9)	2,310 (48)	6,453 (46)

Note

Categories of comorbidity were based on Charlson comorbidity index scores of 0 (no comorbidity), 1 (moderate), 2 (severe), ≥3 (very severe).

^a^ Results are limited to those that residing in Northern Denmark where laboratory data were available: Regions Midt +Nord upon diabetes diagnosis. The population of Northern Denmark is considered representative of the nationwide population.

**FIGURE 2:**
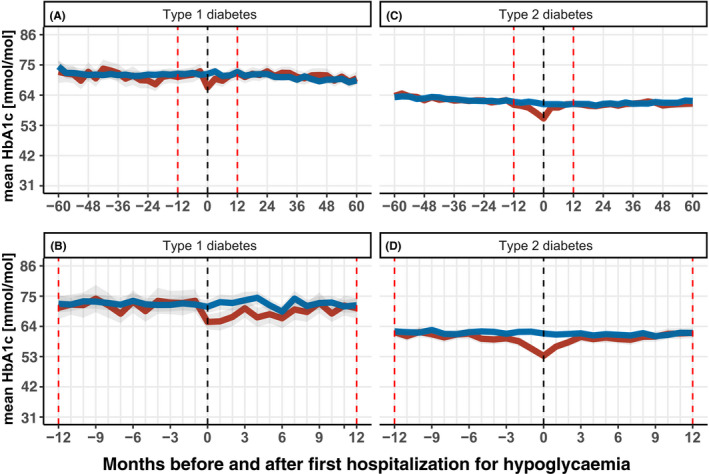
Mean HbA_1c_ (%) before and after first hospitalization for hypoglycaemia. Mean HbA_1c_ 60 months (5 years) before and after first hospitalization (A and C), and 12 months before and after first hospitalization (B and D). Three‐month intervals of HbA_1c_ measurements were used for A and C, and one‐month interval were used for B and D. Red line indicates people with hospitalization for hypoglycaemia, blue line indicates comparisons. LABKA was not complete before 2000, and follow‐up was available 5 years after 2012. For this reason, the figure includes laboratory data from 2000–2017 for people experiencing hypoglycaemia 2005–2012 with type 1 diabetes (n = 409) and type 2 diabetes (n = 1,692) and having laboratory data in Northern Denmark.

Third, we calculated the mean HbA_1c_ with three‐month intervals five years before and five years after first hospitalization for hypoglycaemia (Figure 2: top, [Supplementary Table S1]). The analysis was limited to 1) individuals residing in Northern Denmark where laboratory data were available and 2) individuals hospitalized at a time when laboratory data five years before and after hospitalization were available (ie 2005–2012). Thus, HbA_1c_ samples taken from the event until three months after were depicted at time 0. We similarly computed 12 months before and 12 months after the event using one‐month intervals (Figure 2: bottom). Next, we repeatedly calculated the proportion receiving different glucose regimens separately for type 1 diabetes and type 2 diabetes on a 3‐month basis during one year before and one year after hospitalization for hypoglycaemia. Each individual with diabetes contributed once during a given three‐month period, using data on all redeemed prescriptions during that period. This analysis was performed using prescription data 1996–2017 (ie for people admitted 1997–2016).

The statistical analysis was carried out using R version 3.3.2, and graphics were created using ggplot2 R package version 2.2.1. The project was approved by the Danish Data Protection Agency (file number 2014–54–0922).

## 3 RESULTS

### 3.1 SIRs of hospitalization for hypoglycaemia

The overall SIR of hospitalization for hypoglycaemia per 100,000 person‐years (Figure 1: A) was 17.7 (95% CI 16.4–19.0) in 1997, rose to 30.3 (95% CI 28.5–31.9) in 2003, remained stable until 2010 and then gradually declined to 22.0 (95% CI 20.8–23.3) in 2017. The SIRs according to age groups are presented in Figure 1: B and C.

### 3.2 Characteristics of individuals with diabetes hospitalized for hypoglycaemia

Table 1 presents the characteristics of 3,479 people with type 1 diabetes and 15,329 with type 2 diabetes hospitalized for hypoglycaemia during 1997–2017 and matched comparisons. Median age of people with type 1 diabetes at time of hospitalization for hypoglycaemia was 30.8 years (IQR 18.4–41.9), 42% were female, and 18% had non‐diabetes comorbidities. Median age of people with type 2 diabetes at time of hospitalization was 73.7 years (IQR 63.6–81.5), 46% were female, and 66% had comorbidities. The matched comparisons consisted of 10,419 people with type 1 diabetes and 46,009 people with type 2 diabetes. In the Northern Denmark subcohort, median HbA_1c_ at time of hospitalization was 66 mmol/mol/8.2% (IQR 57–77 mmol/mol/7.4–9.2%) in type 1 diabetes (Table 1, bottom). Individuals with type 2 diabetes and hospitalization for hypoglycaemia had a median HbA_1c_ of 55 mmol/mol/7.2% (IQR 46–69 mmol/mol/6.4–8.5%).

### 3.3 HbA_1c_ before and after hospitalization for hypoglycaemia

People with type 1 diabetes and hospitalization for hypoglycaemia had a stable HbA_1c_ from five years to one month prior to the hypoglycaemic event (Figure 2: A). HbA_1c_ was at 73 mmol/mol (8.8%) one month prior to the event and declined to an HbA_1c_ of 64 mmol/mol (8%) at the time of hospitalization, corresponding to a reduction of ~12% (Figure 2: B). Twelve months after the event, HbA_1c_ gradually increased to 70 mmol/mol (8.5%). The HbA_1c_ of comparisons was stable over time. In the people with type 2 diabetes and hospitalization for hypoglycaemia, HbA_1c_ gradually decreased beginning six months before the event from 62 mmol/mol (7.8%) to 53 mmol/mol (7%) at time of the event, corresponding to an overall reduction of ~15% (Figure 2: D). Within twelve months after the hypoglycaemic event, HbA_1c_ had returned to the starting point of 62 mmol/mol.

### 3.4 Glucose‐lowering drug treatment before and after hospitalization for hypoglycaemia

**FIGURE 3:**
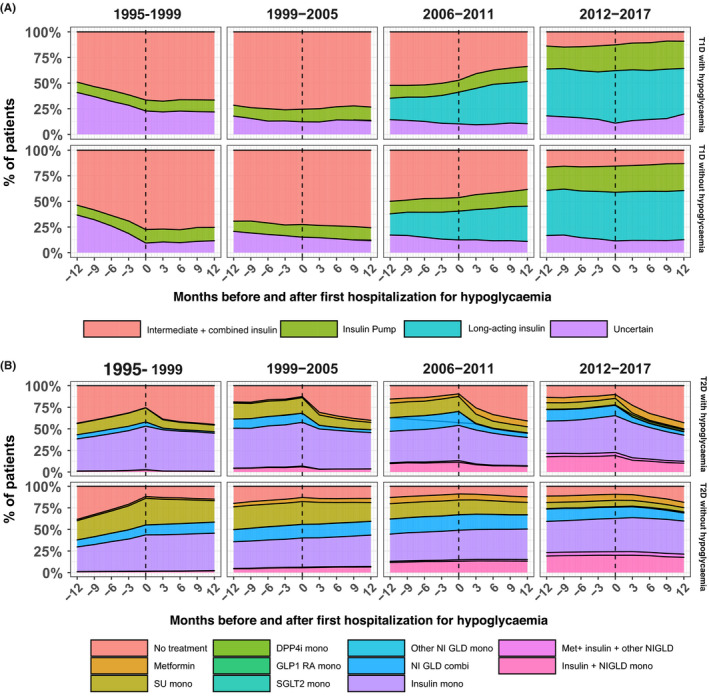
Insulin treatment and glucose‐lowering drugs before and after first hospitalization for hypoglycaemia. Proportional view of people with type 1 diabetes (T1D) (n = 3,479) in panel A and type 2 diabetes (T2D) (n = 15,329) in panel B with first hospitalization for hypoglycaemia and comparisons receiving different types of insulin regimens and glucose‐lowering drugs 12 months before and 12 months after first hospitalization. The dotted line indicates time of first hospitalization for hypoglycaemia.

People with type 1 diabetes and hospitalization for hypoglycaemia appeared to receive similar insulin treatment regimens as the type 1 diabetes comparisons, with similar dynamic tendencies in insulin therapy preceding and after the event date (Figure 3: A). The predominant treatment therapies in people with type 2 diabetes and hospitalization for hypoglycaemia were insulin monotherapy, insulin combined with one glucose‐lowering drug and SU monotherapy (Figure 3: B). After hospitalization, a clear trend in drug discontinuation was present with 45% of people with type 2 diabetes receiving no treatment one year after the event. Overall, people with type 2 diabetes and hospitalization more often received insulin‐based regimens (either monotherapy or insulin combined with one glucose‐lowering drug) (55%) than did comparisons (45%). Furthermore, people with type 2 diabetes and hospitalization for hypoglycaemia appeared to receive less SU monotherapy (10%) compared with comparisons (20%).

## 4 DISCUSSION

In our study, we recorded a gradual increase in population‐based SIRs of first hospitalization for hypoglycaemia in Denmark from 1997 to 2003 followed by a stable period till 2010 and succeeded by a decline to 2017. We observed a similar HbA_1c_ reduction in the months preceding hospitalization for hypoglycaemia in people with type 1 and type 2 diabetes. To our knowledge, this is the first population‐based study investigating HbA_1c_ trends before and after hospitalization for hypoglycaemia in a real‐world setting.

The overall inclining incidence rates in our study observed from 1997 to 2003 resemble the findings from the Retrospective Cohort Study from UK examining type 1 diabetes and type 2 diabetes from 1998 to 2013.^4^ The authors reported a subtle incline in incidence rates of hospitalization for hypoglycaemia in adults. A subgroup analysis revealed declining incidence rates in people with type 2 diabetes older than 64 years during 2009–2013. In contrast, our incline appears to be driven mainly by the age group 70+ years. The UK study only analysed inpatient data and included a mix of both first and subsequent hospitalization, whereas we were able to confidently identify first hospitalization, which partly could explain the discrepancy. Our results of declining rates during 2010–2017 are supported by one other UK study examining SIRs from 2005 to 2014 ^5^ and two Danish studies reporting decreasing incidence rates of hospitalization for hypoglycaemia in type 1 diabetes adults from 2006 to 2012 ^6^ and in children from 1996 to 2014 ^7^ (though none limited to first hospitalization). Our data on children are also supported by findings from a German/Austrian study reporting decreased rates of severe hypoglycaemia 1995–2009.^19^

These encouraging findings are present despite an increasing diabetes prevalence and are likely caused by a number of improvements in the diabetes management: i) increased awareness on more individualized glycemic management to balance between hypo‐ and hyperglycaemia,^20^ ii) increased availability of technological devices, that is insulin pumps ^2^ and glucose monitoring devices,^21^ iii) increased availability of glucagon treatment outside hospitals and iv) the introduction of newer glucose‐lowering drugs with low iatrogenic hypoglycaemia risk ^22^: GLP‐1RA were introduced in 2005, DPP4i in 2006 and SGLT2i in 2012.^23^ Interestingly, the follow‐up of the original Tayside study in Scotland ^8^ showed that while the incidence of severe hypoglycaemia declined, the total number of events treated was higher due to an increased prevalence of diabetes.^24^ A similar tendency was observed in our data for the entire Danish population. From the year 1997 to 2010, we observed a gradual rise in numerical events of hospitalization (from 801 to 1398 yearly events), and from 2010 to 2017, we recorded a stable amount of events (1100–1200 yearly).

The association between HbA_1c_ levels and the risk of hypoglycaemia is a discussed issue: Zhong et al ^25^ and Lipska et al ^26^ concluded that having high (>75 mmol/mol) or low (<42 mmol/mol) HbA_1c_ in type 2 diabetes was associated with a higher risk of hospitalization for hypoglycaemia. However, other studies have not found this u‐shaped relationship ^27^ emphasizing several other factors need to be taken into account when addressing the risk of severe hypoglycaemia including diabetes type, age, diabetes duration, c‐peptide status and lifestyle.^28^ In line with this concept, we found that people within all HbA_1c_ categories experienced hospitalization for hypoglycaemia. The reason we have included data both on children and adults was to present a broader view on the impact of hospitalization for hypoglycaemia in Denmark. Nonetheless, there are substantial differences between the groups including treatment regimes and c‐peptide status affecting the risk of severe hypoglycaemia. We further investigated HbA_1c_ trends before and after hospitalization. People with type 1 diabetes and type 2 diabetes experienced a similar reduction in HbA_1c_ preceding hospitalization. This could imply an iatrogenic focus on HbA_1c_ reduction in some people, while other episodes are caused by spontaneous errors in self‐care. In the period following hospitalization, we observed gradual one year HbA_1c_ increases in both diabetes groups. In type 2 diabetes, this coincided with discontinuing treatment following the event. Another contributing factor to increasing HbA_1c_ could be the fear of hypoglycaemia causing some people to intentionally reduce insulin doses and discontinuing treatment.

We showed that people with type 1 diabetes and hospitalization received similar insulin regimens as type 1 diabetes comparisons and found no clear treatment differences before and after the event. This important finding reduces the chances of uncovering a specific insulin treatment regimen as an important contributor to hospitalization for hypoglycaemia. It is beyond the scope of this study to investigate changes within each treatment regimen, though alterations in insulin dosage could be an important factor to take into account. Other studies have found irregular adherence to insulin therapy, irregular eating and physical activity alterations as possible contributors to non‐severe hypoglycaemic events,^29^ and the same may well be true for severe hypoglycaemia. The overview of glucose‐lowering drug treatment in type 2 diabetes revealed the proportion receiving insulin therapy increased preceding the hypoglycaemic event, possibly implying an intensified treatment in some of these individuals and implies good monitoring is important following intensified pharmaceutical therapy.

Of all severe hypoglycaemic events, few are hospitalized. Accordingly, most severe events are treated at home and prehospital ^30^ and thus not included in our study, although they can be as serious as the ones resulting in hospitalization.

Study strengths include large population‐based data sources with virtually no loss to follow‐up. We combined prescriptions and hospital diagnosis to identify diabetes diagnosis and time of diagnosis, an approach with documented high predictive value and sensitivity.^14^ Limitations should be noted. We prioritized high specificity when defining type 1 diabetes, that is, high certainty that persons classified as type 1 diabetes had true type 1 diabetes. Some people with type 1 diabetes are diagnosed after age 30 years and would be considered type 2 diabetes in this study. Likewise, individuals with type 1 diabetes who were older than 30 years already in 1977 when DNPR data became available would be classified as having type 2 diabetes in our study (but would not be included in the incident hypoglycaemia analysis if they had an incident hypoglycaemic event between 1977 and through 1996, thus limiting the impact of such misclassification). A recent study ^31^ found that 21% of insulin‐initiating individuals after the age of 30 with register‐classified type 2 diabetes based on their debut age and instead had type 1 diabetes. As people with type 1 diabetes (insulin‐treated) are more prone to experience hypoglycaemia compared with non‐insulin‐treated, this could led to differential misclassification and may slightly affect HbA1c‐ and glucose‐lowering drug trends in the group characterized as likely type 2 diabetes patients. We observed ~10% of people with type 2 diabetes apparently receiving no glucose‐lowering treatment yet experiencing an event. This may partly be caused by people having redeemed and stockpiled drug prescriptions prior to the observation window and thus being falsely classified as non‐treated. This may occur non‐differentially both in patients with hospitalization for hypoglycaemia and comparisons and does not impact our ability to assess differences between groups. Still, in some cases the hypoglycaemic event may have happened related to other causes (eg alcohol consumption).

## 5 CONCLUSION

The population‐based incidence rate of first hospitalization for hypoglycaemia in Denmark has declined by one fourth the last decade. In both diabetes groups, a clearly observable HbA_1c_ decrease forecasts first hospitalization, and profound glucose‐lowering drug discontinuation occurred among people with type 2 diabetes after hospitalization.

## CONFLICT OF INTEREST

All authors have completed the ICMJE Uniform Disclosure at http://www.icmje.org/coi_disclosure.pdf (available on request from the corresponding author) and declare that they received no support from any organization for the submitted work; no financial relationships in the previous three years with any organizations that might have an interest in the submitted work; and no other relationships or activities that could appear to have influenced the submitted work. The Department of Clinical Epidemiology, Aarhus University Hospital is a member of The Danish Centre for Strategic Research in Type 2 Diabetes (DD2), supported by the Danish Agency for Science (grant nos. 09–067009 and 09–075724), the Danish Health and Medicines Authority, the Danish Diabetes Association and an unrestricted donation from Novo Nordisk A/S. Project partners are listed on the website www.DD2.nu. The Department of Clinical Epidemiology, Aarhus University Hospital participates in the International Diabetic Neuropathy Consortium (IDNC) research programme, which is supported by a Novo Nordisk Foundation Challenge programme grant (Grant number NNF14SA000 6). The Department of Clinical Epidemiology is involved in studies with funding from various companies as research grants to (and administered by) Aarhus University. None of these studies have relation to the present study.

## AUTHOR CONTRIBUTIONS

J.S.K., M.B.B. (first author), N.M. and R.W.T. designed the study. J.S.K., M.B.B. and R.W.T. collected the data. J.S.K. and M.B.B. conducted the statistical analyses. J.S.K., M.B.B. (first author) and R.W.T. designed the figures. J.S.K., M.B.B. (first author) and R.W.T. wrote the manuscript. N.M. and M.B.B. reviewed and edited the manuscript.

## ETHICS APPROVAL

Not needed for purely registry‐based studies in Denmark.

## PATIENT INVOLVEMENT

Patients were not involved in setting the research question, the outcome measures or the design or implementation of the study. There are no plans to involve patients in dissemination of the results.

## TRANSPARENCY

The senior author, RWT, affirms that the manuscript is an honest, accurate and transparent account of the study being reported; that no important aspects of the study have been omitted; and that any discrepancies from the study as planned (and, if relevant, registered) have been explained.

## COPYRIGHT

The corresponding author has the right to grant on behalf of all authors and does grant on behalf of all authors, to the Publishers and its licensees in perpetuity, in all forms, formats and media (whether known now or created in the future), to i) publish, reproduce, distribute, display and store the Contribution, ii) translate the Contribution into other languages, create adaptations, reprints, include within collections and create summaries, extracts and/or abstracts of the Contribution, iii) create any other derivative work(s) based on the Contribution, iv) exploit all subsidiary rights in the Contribution, v) include electronic links from the Contribution to third party material wherever it may be located and vi) licence any third party to do any or all of the above.

## Supporting information

Table S1

## Data Availability

Danish law does not allow researchers to share raw data from the registries with third parties. Data can be accessed by researchers through application to the Danish Data Protection Agency and the Danish Health Data Authority.
