# Supplementary material for: Hospitalization for hypoglycaemia in people with diabetes in Denmark, 1997–2017: Time trends in incidence and HbA1c and glucose‐lowering drug use before and after hypoglycaemia
Source: Endocrinol Diabetes Metab. 2021 Jan 21;4(3):e00227. doi: 10.1002/edm2.227 (PMC8279622; doi:10.1002/edm2.227)
Supplement: Supplementary file 1 — Table S1 [file EDM2-4-e00227-s001.docx]

**Supplementary Table 1: Periods and people with diabetes involved in study, an overview.**

| **Content** | **Start** | **End** | **Rationale/comment** |
| --- | --- | --- | --- |
| **Registry** |  |  |  |
| The Danish National Prescription Registry | 1994 | 2017 | Complete nationwide coverage since 1995. |
| The Danish National Patient Registry (DNPR) | 1977 | 2017 | Complete nationwide coverage. ICD-8 until the end of 1993 and ICD-10 thereafter. |
| The Clinical Laboratory Information System (LABKA) database | (1985) Complete since early 2000s | 2017 | Covers Northern Denmark (Regions Midt and Nord). |
| The Danish Civil Registration System (CRS) | 1968 | 2017 | Complete nationwide coverage. Daily updates. |
| **Periods in study** |  |  |  |
| People with diabetes | 1977 | 2017 | We used all available prescription data to identify people with diabetes and to classify diabetes type (CRS). First glucose-lowering drugs prescription redemption defined the population in our our database. Using hospital codes for diabetes will significantly underestimate the prevalent population and poorly distinguishes diabetes types. |
| First for hypoglycaemia | 1977 | 2017 | In our database among ever glucose-lowering drugs initiators we identified first hospitalisation for hypoglycaemia event dating back to 1977 (to avoid skewing of our data, when first identifying people with diabetes since 1994.) |
| Table 1: top | People with diabetes with hospitalisation for hypoglycaemia event from 1997 | 2017 | Nationwide. Upon characterizing the people with diabetes we allowed for a run in period 1994-1996 to make sure temporarily non-adherent people were not included as new people with diabetes. (prevalent people thus had 2-3 years to redeem a prescription). |
| Table 1: bottom | People with diabetes with hospitalisation for hypoglycaemia event from 1997 | 2017 | Restricted to those living in Northern Denmark at time of hospitalisation for hypoglycaemia. Laboratory data are only available in this region. Otherwise same considerations as Table 1:top |
| **Figure 1** | Includes first hospitalisation for hypoglycaemia rates from 1997 | 2017 | Among people living in Denmark first ever hospitalisation for hypoglycaemia event since 1977 was calculated but depicted 1997-2017 |
| **Figure 2** | First hospitalisation for hypoglycaemia event 2005 | First hospitalisation for hypoglycaemia event 2012 | We examined HbA_1c_ five years prior to and following hospitalisation for hypoglycaemia. LABKA was not complete before 2000, and follow-up was available 5 years after 2012. For this reason the figure includes laboratory data from 2000-2017 for people experiencing hospitalisation for hypoglycaemia 2005-2012. |
| **Figure 3** | hospitalisation for hypoglycaemia event 1997 | hospitalisation for hypoglycaemia event 2016 | We examined drug use one year prior to and following hospitalisation for hypoglycaemia. People contributed exactly once every period of observation. Prescription data was complete from 1995. The picture includes prescription data 1995-2017 and people experiencing first hospitalisation for hypoglycaemia 1996-2016. |
